# The goals and selection criteria of sports scholarships: mutual benefits perceived by South African schools'

**DOI:** 10.3389/fspor.2025.1574442

**Published:** 2025-08-25

**Authors:** Christopher Ekron, Brendon Knott, H. Thomas R. Persson, Mogammad Sharhidd Taliep

**Affiliations:** ^1^ Department of Sport Management, Centre for Sport Business and Technology Research, Cape Peninsula University of Technology, Cape Town, South Africa; ^2^ Department of Sports Sciences, Malmö University, Malmö, Sweden

**Keywords:** sports scholarship, selection criteria, elite school, high school, South Africa

## Abstract

In South Africa (SA), the legacy of apartheid has resulted in significant disparities within the nation's education system. Elite schools may be public or private, offering a superior educational and sporting environment that is unaffordable for most of the population. However, it is common for these schools to offer sports scholarships to deserving recipients. The Social Exchange Theory (SET) can be used as a theoretical lens to understand the mutually beneficial nature of the relationship between the school and the sport scholarship recipient. While recipients gain educational and sporting opportunities, schools benefit from the athletic talent and boosts their competitive sporting edge and prestige status. Previous studies have considered the progression of the scholarship recipients. However, none have undertaken to explore a more nuanced understanding of the schools' perspective. This qualitative exploratory study, therefore, aimed to reveal the goals and selection criteria for sports scholarships among selected elite boys' high schools in SA. Eight in-depth interviews were conducted with senior staff members closely involved in the scholarship process from four elite boys' schools in the Western Cape, SA. The transcribed interviews were thematically analysed to understand the goals and selection criteria. The findings indicate that there are multifaceted goals for the schools. Beyond boosting the schools' sporting talent and achievement, the goals also included fostering diversity and nurturing holistic development among student-athletes. The selection criteria varied between schools, with criteria beyond sporting potential playing a key role, such as academic aptitude, character, consideration of the financial status of the applicant, and the financial impact on the school. The study highlights that sports scholarships in elite South African schools represent a complex interplay between opportunity and expectation, aligning with the SET lens. While scholarships serve as powerful tools for fostering individual growth and institutional excellence, their implementation requires careful consideration of social integration, financial sustainability and equitable access.

## Introduction

### South African school system

During the apartheid era (1948–1994) in SA, one of the distinctive features of the educational system was its organisation and separation along racial lines (1). Schools were separated by race with a disproportionate distribution of resources and opportunities, favouring schools for the white population (2–4). For example, black African schools received almost ten times less funding than white schools (5). The accumulated effects of this funding for white schools have resulted in impressive infrastructure, excellent facilities, sports programmes and highly qualified teachers (6, 7). In contrast, non-white schools are generally under-resourced, often dysfunctional in academic performance, and suffer from a shortage of qualified teachers, little or no sports programmes and high-student-to-teacher ratios (6, 8).

Although the formal desegregation of schooling was formalised post-apartheid, the enduring effects of inequality still linger and are likely to do so for decades if not generations (9). Today, the schooling system is divided into Quintile systems according to the unemployment and literacy rate in the community where the school is situated (10). Schools are ranked between Quintile 1 (poor and impoverished school) to Quintile 5 (wealthy and affluent school) (10). The absence of a dedicated budget for school sports in lower quintile schools paints a bleak picture, as most funding is designated for essential learning materials (51). In contrast, the high Quintile schools can leverage large amounts of private funding through parents, alumni (Old Boys' Unions) and private contributions (6). Despite the significant advancements in equalising education, public schooling in SA consists of two tiers: one privileged and well-resourced and the other poor and under-resourced (11, 12).

Privileged or elite schools are renowned for offering superior education alongside numerous sporting facilities, coaches, and equipment, supported by a longstanding tradition of sporting excellence and opportunities (12). It is important to note that the literature does not provide a singular, definite definition of elite schools. They are broadly described as “primary and secondary schools of very high rank” (13). According to Khan (14), elite schools are distinguished by their disproportionate control over or access to a wide range of relevant resources, which include academic capital, social ties to elite families and other institutions of power, the capacity to guide and transfer culture, and considerable economic capacity.

### Sport schools

Many elite schools in SA have traditionally been boys' schools that greatly emphasise sports. These elite boys' schools have produced a disproportionately large number of elite athletes at a national level. For example, most of SA's international male cricket and rugby players come from these elite boys' schools (15, 16). Local and international tournaments, cash prizes and live streaming of matches, with thousands of spectators are common at these schools (17). Winning promotes school pride, is closely tied to the school's tradition and impacts the school's reputation (18). However, there are only a limited number of these schools in the country, and competition to enter them is great (7).

Schools with a strong sports profile are neither new nor unique to SA. In many parts of the world, sports schools mushroomed in the 1990s (19, 20). A common denominator for these sports schools is that they have an athlete development programme to pursue sporting excellence while maintaining high academic standards (21, 22). The demand for entry into these prestigious schools has standardised selection and admission requirements (23), often formulated by sports federations (24). These institutions exhibit a predisposition in favour of gifted athletes and those possessing the potential to succeed in their sport, resulting in talent identification and selection procedures that prioritise specific abilities and personalities, culminating in relatively homogenous talent pools (22). Recent literature highlights the increasing intensity of youth sports programmes, underscoring the need for a comprehensive approach to holistic athlete development across all facets, including academic, athletic/physical, psychosocial and psychological (25, 26).

### Sport scholarships

The provision of sports scholarships in SA is one avenue for children from disadvantaged backgrounds who excel in sports to gain access to these elite boys' schools, which would otherwise remain inaccessible due to financial constraints (27, 28). Sport Scholarships are an educational structure that provides talented students with an opportunity to attend a school with the necessary infrastructure, teachers, and coaches to assist them in excelling in their personal development while also getting a good education (18, 27–29). Most elite boys' schools in the Western Cape, SA, offer sports scholarships to talented athletes to enhance their sporting programmes.

In a South African school environment, scholarships are generally provided for high school students, often identified as 12 years old (Grade 6) and awarded in Grade 7 for their first year in secondary school, which is Grade 8. Furthermore, talent scouts or recruitment teachers attend tournaments and festivals to identify gifted youth athletes, who are then offered sports scholarships to these elite schools (18). However, selecting sporting talent at a young age has been proven a difficult task (30, 31), with few elite juniors later achieving an equivalent competition level at a senior age (32).

### Theoretical framework

Moffatt (18) raises a pertinent question: Who benefits from the scholarship, the school or the recipient? Feldman and Wallace (33) stress that students gain social and cultural capital by attending elite schools. Hobden and Hobden (34) state that students are well-prepared for life and academic studies, developing resilience. While sports scholarships benefit the individual recipient, the benefits also extend to the school. For example, elite schools can diversify their student demographic profile and attract top talent (12). Thus, schools may use scholarships as a marketing strategy (35), wherein learners become valuable assets, especially those who exhibit talent in sports (36). With school sports competition intensifying, scholarships awarded to promising youth athletes raise the school's sports profile (18). Consequently, offering scholarships has become crucial to the school's overall success.

However, in the context of this study, the focus is not the exchange cost in terms of R, $, or €, but the schools' views. Hence, a comprehensive theoretical framework must enable an analysis of non-economic, social and economic exchange. Although the sport value framework (SVF) (37) stands out as a potential candidate, mainly because it allows for an argument of where the sum is greater than the parts, i.e., it is never just about the exchange between two parts. However, the SVF lacks the ability to shed light on the giver–receiver relationship in the context of the schools' scholarships. In a similar fashion to SVF, the social exchange theory (SET) is commonly used to look at the relationships involved in the (social) exchanges between two or more parties understood through the lens of reward, cost, opportunity, outcome, transaction, and payoff (gain) (38). Conceived in line with Blau's (39) idea of social exchange limited to actions which are contingent on rewarding reactions from others, i.e., an assumption of reciprocity, SET will serve the purpose of exclusively discussing the schools' views on the exchange.

According to SET, the giver may not be motivated by a one-to-one reciprocity, but one to three or more, is instead opening rather than limiting this study (38). After all, the schools do exist in a common market made up of different actors—stakeholders—that are either affected by or affect the schools' actions. Such stakeholders may be parents/families (of present and potential future students with or without scholarships), previous students, benefactors, sports clubs/leagues/national teams, and the general school system. Who the most relevant stakeholders are may change depending on the aspect discussed but are those which the schools are dependent upon (40).

The mutual benefits of scholarships for both school and recipients (12, 18, 34) provides a rationale for applying the SET to understand the goals and selection criteria for school sports scholarships. The application of SET has been used to discuss various factors in selection processes, such as the study by Czekanski and Barnhill (41), which used SET to consider the recruitment process of college athletes. The SET proposes that when one person does a favour for another, it creates an expectation of gratitude and reciprocity (39). This theory emphasises the importance of intrinsic and extrinsic rewards in social interactions as individuals strive to achieve outcomes they cannot obtain alone (39).

The theory posits that individuals engage in social interactions by weighing up the costs and benefits involved, aiming to maximise rewards and minimise costs. In the case of college or school sports teams, coaches aim to identify individuals who possess unique skills that will improve the team's ability to win competitions, and student-athletes seek a team that values their skill set and provides them with the best opportunity to achieve their athletic and academic goals (41). This indicates that while colleges or schools are looking for the best talent, the individual is also looking for the best environment to maximise their talents. This is particularly relevant in the multifaceted environment of elite boys' schools and competitive sports. SET provides a comprehensive understanding of the dynamics of relationships and interactions and highlights the reciprocal nature of the factors in the sports scholarship selection criteria.

In SA, the micropolitics of admissions into elite schools is complex and enabled by post-apartheid macropolitics (7). The lack of a transparent system for sports scholarships in SA's elite schools contributes further to the already significant divide between well-resourced and under-resourced schools. No previous study has examined these selection criteria for sports scholarships at elite boys' schools, which presents a notable gap in the literature investigating schools' perspectives on sports scholarships. This study aims to gain an understanding of the goals and selection criteria, the schools' views of the exchange benefits for the recipients of scholarships, the schools' reciprocity expectations, the schools' value assessments, and the schools' view on the exchange costs of sports scholarship recipients among elite secondary boys' schools in SA.

## Methodology

### Research approach and paradigm

This study adopted a qualitative research approach to deepen the understanding and clarify the context of the school's goals and selection criteria for sports scholarships. Qualitative research is suited for exploring complex social phenomena and gaining in-depth insights into the perspectives of key stakeholders. Given the focus of this study, an interpretivist paradigm underpins the study, which acknowledges that reality is socially constructed and that meaning is derived from individuals' subjective experiences (42). A constructivist approach was adopted, recognising that school decision-makers shape scholarship selection criteria based on institutional goals, historical influences, and perceived benefits.

### Sampling

Purposive sampling was used to select four elite boys' schools in the Western Cape province that engage in sport scholarships as participants in the study. These schools were selected due to their considerable size (700–1,300 pupils), being well-established (each founded over 100 years ago), and with strong sporting reputations. Furthermore, these schools compete at the highest levels in their major sports and have similar sporting facilities and resources.

The sample consisted of eight participants, with two respondents from each of the four schools who had extensive knowledge of the scholarship process being identified and approached for an interview. The Principal of each school was selected, as well as either a Deputy Principal (in three cases) or a senior staff member. The senior staff member played an active role in the selection and recruitment process. The participants are considered key informants due to their central roles in school management and their ability to provide insight into the study's research problem from the school's perspective.

### Ethical considerations

The researcher obtained permission to interview the participants, who were required to sign a consent form stating that they had accepted the invitation to participate in the study. Participants were informed that their participation in the study was entirely voluntary, that their names would be kept confidential, that no participant would be forced to complete the interview and that they may withdraw at any time. Ethical clearance for the study was granted from the ethics committee at the primary investigator's tertiary institution in SA (Clearance certificate: 2023_FBMSREC_ST09).

The researcher designed a structured interview guide to ensure that interviews remained focused on the topic. The questions were then evaluated by two critical friends in the field of sport development. To validate the interview guide, two pilot interviews were conducted with retired Principals of elite boys' schools, which closely mirrored potential respondents. Based on their feedback, edits were made to the guide to enhance understanding of the questions and improve the flow of the interview. Ten questions were finalised, covering two themes: the school's goals and the selection criteria. Each participant was asked the same set of questions, such as: “What is the main goal in offering a sports scholarship?” “What are the school's selection criteria?” The interview guide was designed to focus on aspects of exchange relationships, such as the perceived benefits and reciprocity expectations.

An essential factor for qualitative research is minimising researcher bias. However, complete elimination of bias is impossible. The lead researcher of this project works as an educator at one of the schools under study and is familiar with its sporting environment, culture, and scholarship programme. However, to minimise bias, the researcher followed the same interview protocol and structured guide for each participant.

The interviews were in-person and were audio recorded. The researcher also took notes during all the interviews. Interview durations ranged from 45–60 min.

Interviews were transcribed verbatim by the researcher as soon after the interview as possible to ensure an accurate reflection. To enhance credibility and ensure the accuracy of the data, the verbatim interview transcripts were returned to participants for their feedback. All the participants agreed that the transcripts were a true reflection of their interviews.

### Data analysis

After listening to the recordings and reading the transcripts, the researcher began the analysis by coding the data. Initially, predefined codes were created based on the literature review. These codes were clustered under two code families, representing this study's objectives (i.e., goals and selection criteria). The SET theoretical framework guided the development of four pre-defined code families, namely: exchange benefits for the scholar, reciprocity expectations for the school, value assessment, and exchange costs. Individual codes were developed inductively and assigned to the code family. For example, the code family “Goals: exchange benefits for the scholar” had the following individual codes: Holistic development, career advancement, and social network. See Table 1 for the full set of code families and individual codes.

**Table 1:** Development of codes.

Code family	Individual Codes
Goals: Exchange benefits for the scholar	Holistic development
	Career Advancement
	Social network
Goals: Reciprocity expectations for the school	Diversity
	Talent
	Sports Achievement
	Inspire/motivate other scholars
	Marketing
Selection criteria: Value assessment	Academic and language proficiency
	Character traits
	Talent identification (speed; agility; coordination; size; skills)
	Sport achievement
	Sport codes
	Race
Selection criteria: Exchange costs	Parental contribution
	Financial costs
	Boarding versus day student

The researchers used ATLAS-TI, a statistical software package, to store the data and to perform the coding process. As the data analysis progressed, no new codes emerged, indicating saturation. To maintain confidentiality, the interviews were labelled 1–8. When respondents mentioned a specific person or school name, it was replaced in square brackets with an adjective.

### Findings

The following sections present the findings in relation to the key emergent code family identified during the interview analysis while critically engaging with SET to interpret the dynamics of sports scholarships in elite South African schools.

### Goals: exchange benefits for the scholar

The participants described the exchange benefits that the scholarship represents for the scholar as a multitude of purposes, which were coded under three items: Holistic development, Career advancement, and Social network development. For example, the quotation below reveals these benefits:

The boy and his family benefit through him, giving him the opportunity to receive an all-around education here, being exposed to playing sports at a high level against high-quality opposition and having the opportunity to be selected for provincial teams, which could advance his potential career. (Participant 2)

Besides providing the student with the chance to practice his sport at a highly competitive level, the scholarship provides a gateway for the student to access a comprehensive educational environment, including opportunities to engage in various activities such as music and theatre, and facilitate access to a broader social network, fostering connections that enrich the educational experience and personal growth. Participant 4 emphasised this when saying:

“We do aim to improve the person, the boy, and to give them a sort of, a bit of future, and to help establish networks, ultimately, for the boy, through being here, to have a future career”.

### Goals: reciprocity expectations for the school

The school's objective in granting scholarships includes encouraging diversity, enhancing the school's sporting reputation through sports achievement, inspiring/motivating other scholars, and promoting the school through marketing.

Although participant 1 explicitly stressed that the main purpose was to diversify their pupil body, all participants emphasised the aspect of diversifying the student body by granting educational access to perceived “underprivileged” students. All are perceived to foster a more inclusive and diverse academic setting for the school.

The school gets the benefit of having the child play at a high level, and so our benefit is that a talented player can lift other boys at the school to and inspire other boys in their team who are not on scholarship. But it can also help with the marketing of the school if your sports teams are performing well. (Participant 2)

Fostering sporting progress. You know, there would be no boy school that does not want a good team…with the understanding that they bring that ability, that skill brings with encouragement to the players around them. (Participant 4)

### Selection criteria: value assessment

Participants mentioned that potential students and their parents find out about the scholarships in various ways. Information on the different scholarships can be found on the schools' websites, but students and parents also find out about the scholarships through word-of-mouth and scouting by coaches or heads of sports. As far as recruitment, the following quotation is revealing:

We also have some, it's not a huge part of the school's environment, but there's some recruitment, I guess, in as much as our head of rugby goes to the school provincial tournament and sees a big player there and talks to the parents and says, would you consider coming to (school), we offer a scholarship that will happen, particularly in cricket, rugby and hockey. (Participant 4)

Additionally, one school hosts a clinic to identify gifted athletes. The process of students and their parents seeking information, along with scouting, generally begins in Grade 6 (at 12 years old), and applications for secondary school (Grade 8) open at the start of the students' Grade 7 year (at 13 years old). It is evident that the schools' recruitment processes are crucial for admitting new scholarship students, suggesting that sporting skills take precedence over academic assessment. Relatedly, two schools mentioned the need to limit excessive scholarship advertising.

I think if we advertise sports scholarships, we will be inundated with sports scholarships and appeals upon appeals if they are denied for whatever reason. Scouting is a massive part of where we are in terms of offering a scholarship. (Participant 6)

Every respondent stated that most scholarship recipients come from the Western Cape and that recruitment focuses on various backgrounds, typically from schools that are not the main feeder schools of regular students. Regarding the geographical aspect, participant 7 stressed that they search for potential candidates in a variety of areas.

I think it is mostly the Western Cape. You must remember that we get a lot of applications from boys from afar. (Participant 7)

Despite the described clear focus on recruiting sports talents, all schools mentioned that sports scholarship students are chosen based on their academic abilities, character traits, and athletic potential. All the participants described the selection process as involving both objective measures, such as academic performance and sports achievements, and subjective assessments, like character evaluations. While it is important to highlight that the schools share common criteria, specific criteria may be of importance in relation to different priorities and funding available.

All participants were asked to indicate their specific selection criteria for academic performance, sports achievements, and character. In terms of character, it was indicated that students' ability to adapt to their new environment is essential. One respondent indicated that they assess “whether he's (the student) the right fit for the school” (Participant 5).

The schools' character assessments for potential students involve interviewing potential students and considering reference letters from previous schools or coaches. This approach allows the school to observe potential students in various contexts. The schools identified that character is key for students who are not from the general feeder schools and that identifying students who can adapt to the new environment is important. Students involved in many areas, such as playing a musical instrument or who were in leadership positions at their primary schools, are positive traits that the schools consider.

We interview and we are looking for boys who we think will fit well into the school. As far as possible, we try and judge their character from any reference letters we get from their previous school or coaches and that would inform our decision. (Participant 1)

All schools mentioned the importance of academic achievement in selecting sports scholarships. However, none indicated a clear or objective requirement. Instead, it appears that they assess whether the students are “academically capable” (Participant 2) and able to cope in a secondary (elite) school academic environment. The following responses emphasise the academic merit considerations:

The student has to be academically capable. We have burnt our fingers in the past. People who are on sports scholarships struggle academically, and if they struggle academically, it just affects everything. (Participant 2).

They don't get accepted if the student is failing…no matter how good the boy is. (Participant 6)

The two important subjects considered essential when considering academic performance are English (“able to converse or understand the English language”) as the medium of instruction and Mathematics, as indicated in the following responses:

They must be able to converse or understand the English language. The student is going to be educated in the language, and so he needs to understand that. (Participant 2)

So, they might have fallouts in terms of language and Maths, which is not reflected in the academic report we get from the student's primary school. (Participant 5)

You know, there are things you can't account for, foundations in mathematics, for instance, that weren't given in primary school; kids have to try and catch up in high school; it's almost impossible. (Participant 4)

Sporting potential was confirmed as the main criterion for awarding a sports scholarship. As participant 3 stated, “I will tell you the number one priority around awarding a sports scholarship is they need to be excellent sportsmen.” The schools identified three sports as their primary sports for awarding scholarships at the school. Rugby, hockey and cricket are the most common, with a few minor scholarships available at one school for water polo, squash, and swimming. Rugby was a consistent scholarship offered at all the schools and was frequently referred to as the school's core sport. All the schools encourage sports excellence, and providing scholarships is one way to ensure that the schools' sports remain competitive. Participants 6 and 8 highlight these aspects in the below quotes.

Because we are recognised as a high-performance rugby school, I think by default, the majority of our scholarship funding goes to rugby boys. (Participant 6)

I think generally, (school) basically gives scholarships based on performance, and I think the rugby environment is probably the most complex of all environments. Most of the scholarships go to rugby. (Participant 8)

Participants also mentioned identifying students who play more than one sport as part of their selection criteria while confirming the challenge of identifying sporting talent potential at as early age as 12 for the scholarship applicants. To help them in their recruitment process, schools use provincial-level tournaments at under 12, but they also sometimes request feedback from the primary school coaches and provincial coaches to assist in identifying the students' sporting potential.

We rely on our sports department, it’s very difficult. It's always incredibly difficult to assess a 12-year-old child and to try and assess potential as opposed to ability, and that's what we try and focus on. So, things like speed and agility and hand-eye coordination are really important. General ball skills, those sort of skills or things, are what we would look for. Interestingly, size is not something we will look for, because size masks, all sorts of things you can get away with being a giant boy playing rugby at under 13, if you don't grow you have no skills then. There's no kind of return from the school on the investment that they've made in the child as a sportsman. (Participant 2)

### Selection criteria: exchange costs

It was made clear that not all scholarships are equal. Overall, participants mentioned that various scholarship amounts are offered to students. According to one school, even if a student's scholarship covers 100 per cent of the tuition fee, he may be eligible for additional benefits from the school, such as laptops, clothing, medical aid, and tour fees. In this case, the scholarship amount is determined before the student enters grade 8. A financial review is conducted with his parents, and extras offered are based on the family's financial needs.

The participants also indicated that tuition fees alone would not contribute to a successful scholarship. One participant stressed the importance of knowing what the student requires during his schooling, that paying only for his tuition is insufficient, and that “extras” are necessary for the student's development.

Initially, paying school fees was the thing, or a portion of school fees. But as the sort of social cohesion element has grown, we've come to realise that there are so many other additional things that are needed. (Participant 4)

Yeah, so I think the thing that schools miss about these things. It's more than just the scholarship it's got to be some care for the kid, and I think that is where we schools get it wrong. But generally, if we look at material things, yes, the kid coming out of the township, you are going to help him with clothes, and you are going to help him with tours. So, it's more than just 100% scholarship, it becomes 120% or 130% scholarship, because you have to do those things. You can't take a guy out of his environment, put him into a school, and he's got to look after himself and can't afford it. So, there's a lot of other things that come with that. (Participant 8)

All schools emphasised the importance of parents contributing in some way because it is supposed to hold parents accountable for their child's educational development and journey, as explained by one participant below.

So, when we hand over any scholarship, we want parents to take responsibility. Because what happens is if we give you everything and your parents know that everything is sorted, the parents are not that heavily invested. (Participant 6)

One participant mentioned finding the “right balance” in the boarding house, and too many top athletes in the boarding house create an elitist atmosphere.

We try to discourage our high-performance boys to all be boarders. Because, yeah, then you create elitism, where the boys feel. Listen, I am here for sport, I get taken care of at the hostel; I get my meals on time and do this and do that. And that becomes problematic within the school. (Participant 6)

## Discussion

By offering a scholarship, the school enters a social exchange with the hope that the rewarding reaction from the recipient is the athletic prowess that the school seeks. Most likely, the student and his family view the scholarship as an opportunity to access quality education, social mobility, and athletic development. From a social exchange perspective (38), these benefits reflect a transactional yet evolving relationship between the student and the school. The student gains access to an elite educational environment, but in return, the school expects contributions in the form of performance.

It could easily be assumed that economic theory on decision making, understanding it as a rational calculation on return, would be the first theory to understand the exchange act of giving a scholarship. This would be to understand the schools' act of giving, followed by reciprocity from the point of view of rational calculation on return on investment. However, when the recipient student is, in fact, a child, not even the starchiest rational choice believer could argue that such a presupposition holds explanatory value in the case of a child. Instead, even though the schools rationally cannot be motivated by expected return, not least due to the relative age effect's impact on talent picking (43), we have the paradox that gift-giving usually brings a return (38). In the context of scholarships, reciprocity depends on “talent” picking, a concept we will revisit.

Many of the respondents pointed out that their school's objective in granting scholarships included encouraging diversity and enhancing the school's sporting reputation. This aligns with Geyer and Walton's (27) findings, which stressed that diversity was particularly significant in the aftermath of apartheid in SA. Although being the one that represents diversity most likely comes with the price of pressure, the schools' willingness to diversify the student body is best understood if we enlarge the concept of reciprocity. If it is significant to show that the school is diversifying its pupil body, then it is appropriate to assume that reciprocity may be greater than the one-to-one relationship between the school and the pupil (and his family). Hence, the calculated reward might be expected to come from elsewhere within the schools' broad network of stakeholders (40).

To specifically grant sport-based scholarships is best understood from the schools' experience of highly competitive sports leagues, cash prizes and television coverage, which come with increasing pressure on the school and coaches (17). Hence, sports scholarships are one route to attract the top talent in the country and maintain the schools' competitive edge in sports, which in turn assists with the school's marketing. Schools offer scholarships to talented and competitive students, aiming to significantly enhance overall sports performance and set higher standards of sporting excellence.

Consequently, scholarships offer elite boys' schools a chance to present themselves as inclusive institutions while also attracting top sporting talent. This situation places students in a position where they must continuously justify their place within the school. The pressure to perform potentially fosters a power dynamic in which students are expected to consistently “repay” the opportunities afforded to them.

Participants indicated that character, academic performance, and sports achievements were key selection criteria. In terms of character, it was indicated that students' ability to adapt to their new environment is essential. The emphasis on “fit” is a contradiction to the mentioned diversity. While the “to adapt” could be read as acculturation, where a newcomer may learn and adapt to the new environment, the “fit” is closer to cultural assimilation, where a newcomer should conform to existing standards and norms by divorcing himself from his cultural background (44, 45). Independent of this, students from different socio-economic and cultural backgrounds may experience pressure to conform to the dominant schools' norms.

In contrast to both the description of “adapt” and “fit”, Shields (46) argues that the aim of education is to promote the development of intellectual, moral, civic, and performance character. This is echoed by Foan et al. (47), where performance and moral character were found to be the most frequently identified factors in identifying rugby union talent. By working from the idea of “adapt” and “fit”, the participants are, in part and most likely unreflectively, disqualifying the quality of the schools in which the boys will become students.

While schools aim for all scholarship recipients to be academically competent, this has not consistently been the case. In some instances, there has been leniency regarding accepting students who achieved poor academic marks, not always to the benefit of the school. This consideration is extended to students from disadvantaged backgrounds. The academic standard of these students might not be commensurate with those of the elite schools offering scholarships. The reason for this is primarily due to the two-tier schooling system in South Africa (11, 12).

This concern provides insight into the potential cost of selecting academically underprepared students. The strain of academically supporting students and bearing the reputational strain if a student fails a grade. Within the lens of SET, it reflects the school's attempt to balance the cost of academic underperformance and the school's reputation with the benefit of sporting excellence. When the cost outweighs the benefit, the school perceives the scholarship as unsustainable and would not accept these students despite their excellent sporting ability. Learners from disadvantaged schools may fail to acquire appropriate foundational literacy and numeracy skills (52). These gaps emerge in primary school and widen progressively toward the end of high school (52). It is reported that without foundational literacy and numeracy, learners struggle in all subjects, leading to high dropout rates (52). It is therefore critical, yet complex, for schools to understand the student's academic standard prior to selection, because inadequate literacy and numeracy competencies would predispose the student to failure later in high school. This highlights the complexities faced by schools in balancing opportunity with preparedness and in supporting, not just selecting, students. Through the lens of SET, the schools assess the potential rewards and costs of scholarship opportunities, often resulting in challenging decisions when foundational academics create disparities in the anticipated exchange.

Interestingly, there are sport school systems elsewhere applying a national and hence more uniform approach to the selection criteria. For example, the selection into the Swedish upper secondary elite sports schools is governed by national standards (23, 48). Students are required to obtain an acceptable minimum pass grade for primary school, with pass grades in Swedish (or Swedish as a second language), English and Mathematics. Similarly, the results obtained in our study show that literacy and numeracy play a key role as entry requirements in Sweden (48). However, because the entry into elite sports schools in SA is not regulated, these schools can opt for more stringent selection criteria. By doing so, it “safeguards” the schools from accepting students who struggle academically, which is a potential reputational risk.

Independent of the academic difficulties mentioned, prioritising sport over other aspects of life may also disadvantage most youth athletes who will never be elite athletes. Hence, academics need to be a critical part of the student's development (49). This aligns with Moffatt's (18) earlier findings that elite South African boys' schools emphasise sports, particularly rugby, and excelling in this sport to promote pride in the school. This is consistent with Lombard (17), who stated that students must be developed on their future potential, not their current sporting performance. This indicates a greater subjectivity in the selection process.

The complex task of identifying talented athletes at a young age, which, apart from an assessment of “speed, agility and ball skills”, is rather subjective. Hence, there is no guarantee that these athletes will fulfil their potential. Thus, while the sporting level is the main criterion for awarding a scholarship, it is clearly acknowledged that assessing and selecting talent at 12 years of age is difficult. For example, only 24% of U13 rugby provincial players progress to play provincial under-18 rugby (50). While this study focuses on the development progression of U12/13 players into high school, talent identification remains challenging for all ages. Güllich et al. (32) further highlights the progression challenges of young athletes, concluding that 82% of international-level seniors did not reach the international level as U17/18 juniors.

The findings of our study highlight the evolving understanding of what a scholarship should entail. As social cohesion has developed, so has the recognition that students require more than tuition fees. These additional costs assist with the integration and development within the school environment and underscore the importance of addressing the holistic needs of the students. From the school's perspective, parental involvement and care are important. If a parent can afford to contribute, they should, as this is believed to foster a sense of responsibility and investment in the child's education, which in turn has a positive effect on the student's development and success. This suggests that it may be perceived as unsustainable or undesirable without some level of parental exchange, even with full financial support. This again relates to the SET theory, where the scholarship represents a reciprocal investment by the school and parents that creates a mutual responsibility to ensure the scholarship's success (39).

By providing a comprehensive scholarship that expands beyond covering tuition fees, the schools attempt to assist in the student's well-being and success and allow the student to participate and thrive fully in the schooling environment. This approach acknowledges the broader socio-economic challenges these students may face and the need for schools to provide holistic support.

While scholarships are intended to integrate students into elite schools, they can also unintentionally reinforce exclusivity. The findings reveal the reciprocal responsibilities of both the school and the students, as well as their families, in creating the environment for a successful scholarship. It further raises critical questions about the social integration of scholarship recipients and whether scholars feel segregated, either as outsiders or as a privileged subset. This could counteract the broader goals of diversity and inclusion.

It also reveals that the schools are still learning what is best to support the scholarship student and are willing to adapt their offerings to support the student or perhaps the school. However, this is also balanced with the financial costs involved in supporting the student and consideration for how the scholarship student may affect the culture and experience of non-scholarship students.

## Conclusion

This paper investigated the goals and selection criteria for sports scholarships entering elite secondary boys' schools in the Western Cape, SA. The findings illustrate the multifaceted dynamics of sport scholarships in elite boys' schools in SA, highlighting their role as both enablers of individual development and instruments of institutional advancement. Grounded in SET (39), the study reveals that these scholarships operate as transactional mechanisms, offering mutual benefits to both students and schools. The results indicate that schools offer sports scholarships for multifaceted reasons. The schools gain enhanced sporting performance, diversity and reputational growth from the sports scholarship student. At the same time, the student gains access to quality education, athletic development and social mobility. The reciprocity inherent in SET underscores the dual expectations placed on scholarship recipients, where students are expected to perform on the sports field and in academics while adapting to the elite school environment.

This study also revealed that South African elite boys' schools do not have clear sports scholarship selection criteria. There are common factors that include academic aptitude, sporting potential, character traits, students from disadvantaged backgrounds, and financial needs. Schools heavily weigh in the benefits of the potential of athletic excellence against the cost of academic underperformance and poor character traits that could jeopardise the reputation, risk, and resource strain.

While there is overlap in the selection criteria for sports scholarships between schools, the final criteria are dependent on each school's distinct mission, vision, and focus. This variability directly aligns with the study's focus on the schools' perspective and reflects how scholarships are used to support institutional goals.

## Limitations

A limitation of this study is that it only explored the schools' perspective and not that of sports scholarship students. To fully grasp the influence of the scholarship in relation to SET, the perspective and experiences should be investigated in future studies.

## Implications for practice

This paper advocates for the development of clear, consistent and context-relevant guidelines for the selection of scholars. A more structured and transparent approach would not only improve fairness and accountability but also enable schools to identify students with greater potential to excel both academically and athletically. Despite this study's focus on the school's perspective, it illuminates the complex considerations and differing goals and expectations that this perspective represents. Ultimately, the researchers aim to indicate practice and policy implications for how schools can improve their selection of scholars, to achieve their stated goals, including supporting the holistic development of the scholar.

## Data Availability

The raw data supporting the conclusions of this article will be made available by the authors, without undue reservation.
